# Optical coherence tomography angiography for the anterior segment

**DOI:** 10.1186/s40662-019-0129-2

**Published:** 2019-02-01

**Authors:** Wen Di Lee, Kavya Devarajan, Jacqueline Chua, Leopold Schmetterer, Jodhbir S. Mehta, Marcus Ang

**Affiliations:** 1 Singapore Eye Research Institute, Singapore National Eye Center, Singapore, Singapore; 2 Eye-ACP, Duke-NUS Graduate Medical School, Singapore, Singapore; 3 Department of Clinical Pharmacology, Medical University of Vienna, Vienna, Austria; 4 Center for Medical Physics and Biomedical Engineering, Medical University of Vienna, Vienna, Austria; 5 Nanyang Technological University, Singapore, Singapore

**Keywords:** Optical coherence tomography angiography, Vascularisation, Cornea, Iris, Anterior segment

## Abstract

Optical coherence tomography angiography (OCTA) is a rapid and non-invasive technique for imaging vasculature in the eye. As OCTA can produce high-resolution cross-sectional images and allow depth-resolved analysis for accurate localization of pathology of interest, it has become a promising method for anterior segment imaging. Furthermore, OCTA offers a more patient-friendly alternative to the conventional invasive dye-based fluorescent angiography. However, conventional OCTA systems are typically designed and optimized for the posterior segment of the eye, and thus using OCTA for anterior segment imaging can present several difficulties and limitations. In this review, we summarized the recent developments and clinical applications in anterior segment OCTA (AS-OCTA) imaging, such as for the cornea, iris, sclera and conjunctiva. We also compared commercially available OCTA systems, discussed the limitations of adapting current OCTA technology for the anterior segment imaging, and proposed possible future directions for AS-OCTA systems. AS-OCTA provides potential for future clinical applications such as diagnosis of corneal and iris pathologies, pre-operative surgical planning, assessment of new anti-angiogenic therapeutics or evaluation of limbal stem cell deficiency. With further development, OCTA for anterior segment imaging in the clinics may become common in the near future.

## Background

Optical coherence tomography (OCT) imaging is a well-established technology that enables non-invasive and rapid in vivo imaging of the eye [1]. Since it was first introduced, OCT imaging has become an integral part of clinical assessment. By applying low-coherence light and measuring the echo time delay of light backscattered from tissue structures, OCT can provide high-resolution three-dimensional structural images, which are useful for pre-operative diagnosis, intra-operative real-time imaging as well as post-operative evaluation of diseases [2]. Structural OCT systems produce poor delineation of blood vessels due to scattering of light [3]. However, with recent improvements in signal analysis, OCT systems are now able to visualize vascular flow [4].

OCT angiography (OCTA) is an emerging technology for imaging ocular vasculature [1]. It works on the concept of low coherence interferometry and analysis of signal decorrelation between consecutive scans, by comparing phase speckle contrast, changes in intensity or variation of the full OCT signal [3, 5, 6]. OCTA is currently used clinically for vascular imaging of the retina, choroid and optic nerve [7–9]. Commercially available systems are designed to visualize retinal microvessels and have been useful in the assessment of pathologies in the posterior segment of the eye, including retinal neovascularisation, retinal artery and vein occlusion, and glaucoma [1, 10]. While OCTA is now commonly utilized for the posterior segment, research on OCTA for the anterior segment is only in its infancy [11].

Anterior segment imaging of the vasculature is useful for a diverse number of clinical applications, ranging from diagnosis to monitoring treatment of corneal pathologies [2, 6, 12]. Currently, assessment of anterior segment vasculature is limited to slit-lamp photography (SLP) and dye-based angiography. SLP is the most common method to capture the anterior segment vasculature for clinical and experimental applications [4]. However, SLP has limited visualisation of vessels in the presence of corneal oedema, deposits or scars. Thus, image analysis often results in underestimation due to poor sensitivity to smaller vessels and interference from background iris vessels [4, 13]. Also, only two-dimensional information of the vasculature can be derived [13].

Fluorescein angiography (FA) and indocyanine green angiography (ICGA) are more reliable methods for evaluating normal and diseased vessels clinically [1, 4]. It has been demonstrated that these techniques show better vessel delineation than SLP, especially for vessels beneath corneal scars [1, 13]. In addition, leakage observed in FA and ICGA can give information on vessel maturity while differentiating afferent and efferent vessels [1]. Furthermore, since ICG is a large molecule that remains in vessels for long periods, ICG leakage is likely indicative of a pathological condition [8, 14]. However, these invasive techniques are rarely performed due to infrequent but severe adverse reactions associated with the dyes, including gastrointestinal side effects and anaphylactic shock, even for patients with no risk factors or history of allergies [12, 13]. Patients who are pregnant or have impaired liver and kidney function are also not compatible with such techniques [1, 8]. In addition, leakage may prevent visualization of deeper vessels, causing underestimation of the extent of vascularisation [8]. While current angiography methods allow qualitative assessment of the anterior segment vasculature, objective and quantitative evaluation is challenging. Furthermore, as anti-angiogenic therapeutics are developed, new non-invasive imaging techniques that can quantitatively measure changes in anterior segment vasculature are needed [6]. As such, research in OCTA for anterior segment imaging has been garnering attention and importance.

OCTA has many potential advantages over current anterior segment imaging techniques. Firstly, OCTA can rapidly acquire images in a non-invasive and dye-free way, thus avoiding dye-related side effects and offering a more patient-friendly alternative to fluorescence angiography [6]. The absence of leakage also ensures that deeper vessels are not obscured [3]. Secondly, OCTA can produce high-resolution cross-sectional images, which can be segmented into different layers, allowing visualization of vessels at different depths [3]. Moreover, the *en face* mode of OCTA produces C-scans that are oriented from the frontal plane to give an overview of the corneal pathology, which was previously not possible with B-scans [14]. These features can provide accurate localisation of the pathology, which is helpful during planning for surgery or treatment [1, 8]. Thirdly, OCTA has been shown to detect vascularisation even in cases with severe corneal opacification, which would not have been visible with SLP [10]. Lastly, OCTA has only a slight learning curve and can be performed by trained technicians. This provides a more cost-effective method over invasive angiography, which is time consuming and requires a certified clinician to perform the procedure [4, 12]. Nonetheless, it is also important to note the current limitations of OCTA. This includes restricted field of view, lack of information on flow speed, projection and motion artefacts caused by scattering and lack of motion tracking system, inability to differentiate afferent and efferent vessels and the need for careful examination of artefacts that might be mistaken as vessels, such as from hyper-reflective structures like corneal fibrosis [1, 3, 6].

The aim of this review is to summarize current developments in adapting OCTA for anterior segment vasculature imaging, including the cornea, iris, sclera and conjunctiva. We also evaluate the different OCTA systems that are available and discuss potential future directions and clinical applications of OCTA for anterior segment of the eye.

## Review

### Anterior segment optical coherence tomography angiography technology

**Fig. 1:**
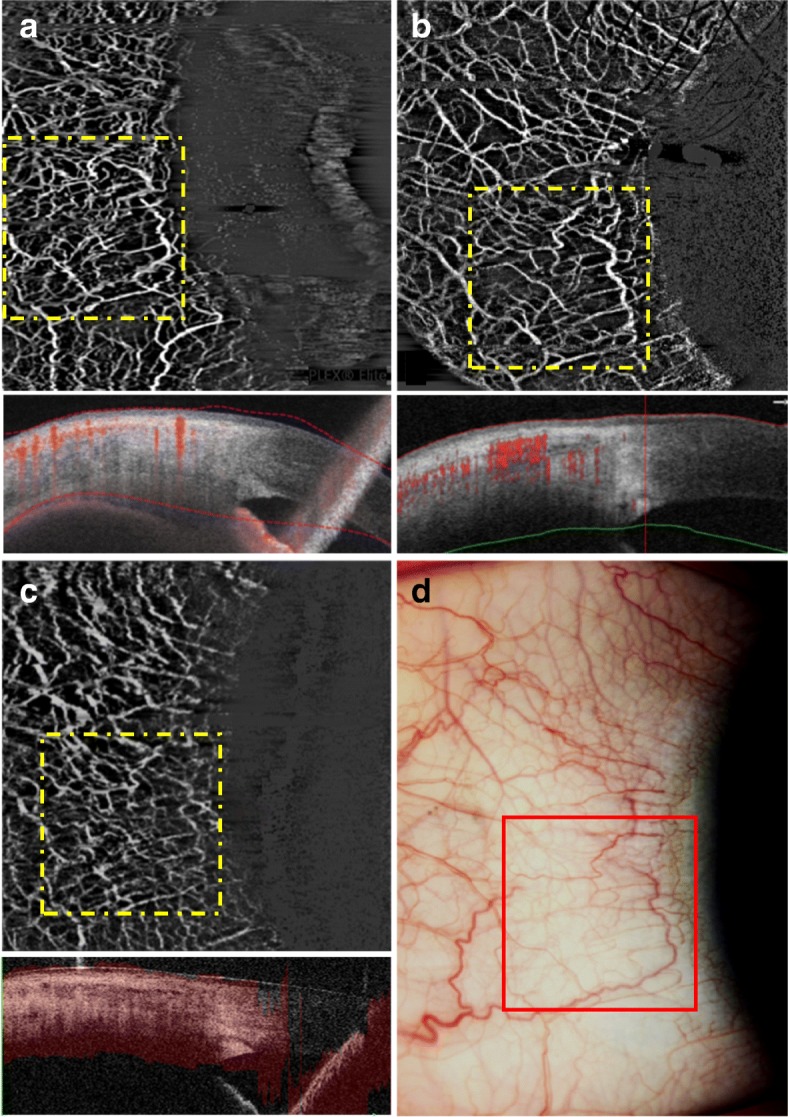
Examples of corneal limbal AS-OCTA scans in a normal healthy eye. We used three different AS-OCTA systems: **a** PLEX Elite 9000 swept-source OCTA system, **b** AngioVue RTVue XR Avanti OCTA system, and **c** Angioscan RS-3000 Advance OCTA system. The corresponding area imaged with slit-lamp photography (**d**) and the OCTA systems are matched (bordered by red and yellow squares, respectively)

OCTA utilizes phase variations, differences in signal amplitude or changes in full OCT signal in consecutive B-scans to detect blood flow [4]. As current OCTA systems are designed for retinal imaging, adaptor lens is needed to image the anterior segment [2, 4, 8]. Current systems use different algorithms to produce images, including full- or split-spectrum amplitude decorrelation angiography (FSADA or SSADA, respectively), optical microangiography and ratio analysis [6]. In addition, these systems also differ in scanning speed, scan area, resolution and other internal software that allow for motion correction, projection artefacts removal or automated segmentation, to name a few. A good image generally requires a good balance between sampling density, field of view and number of B-scans [3]. Oversampling will improve the quality of images but will increase the risk of bulk motion artefacts. In addition, as lateral resolution depends on the spot size of the beam and oversampling ratio, a larger field of view will result in lower lateral resolution, implying that smaller vessels may not be detected for larger scan areas compared with smaller scan areas [13]. Furthermore, each measurement takes about 3 to 6 s and the area of the eye that can be scanned in this duration depends on the A-scan rate of the system, which in turn will affect the resolution of the images obtained [3]. We have briefly summarized the currently available OCTA systems that may be used for imaging the anterior segment of the eye (Fig. 1) along with their corresponding differences (Table 1).

**Table 1:** Comparison of currently available OCTA systems for imaging the anterior segment of the eye

	AngioVue RTVue XR Avanti	Angioscan RS-3000 Advance	Triton Prototype DRI-OCT (swept source)	PLEX Elite 9000 Swept source OCT
Imaging company	Optovue, Fremont, California, USA	Nidek, Gamagori, Aichi, Japan	Topcon Corporation, Tokyo, Japan	Carl Zeiss Meditec, Dublin, California, USA
Algorithm	Split-spectrum amplitude-decorrelation angiography (SSADA)	Complex difference (full spectrum amplitude)	OCTA-Ratio Analysis (full spectrum amplitude)	Complex optical microangiography (OMAGc)
Type of algorithm	Amplitude	Amplitude + Phase	Amplitude	Amplitude + Phase
Light source	840 nm	880 nm	1050 nm	1050 nm
Scanning speed	70,000 scans/sec	53,000 scans/sec	100,000 scans/sec	200,000 scans/sec
Scanning volume	304 × 304 A scans	256 × 256 A scans	320 × 320, 512 × 512 A scans	300 × 300 A scans (3 × 3 mm), 500 × 500 A scans (6 × 6 mm, 9 × 9 mm, 12 × 12 mm)
Scan area (macula)	3 × 3, 6 × 6, 8 × 8 mm	3 × 3 to 9 × 9 mm (12 × 9 panorama)	3 × 3, 4.5 × 4.5 mm 6 × 6, 9 × 9 mm	3 × 3 mm, 6 × 6 mm, 9 × 9 mm, 12 × 12 mm (15 × 9 mm panorama)
Optical Resolution (Axial)	5 μm	7 μm	8 μm	6.3 μm
Optical Resolution (Lateral)	15 μm	20 μm	20 μm	20 μm
Scan duration	3–4 s	5–6 s	4–5 s	Variable (scanning stops when motion is detected)
Axial imaging depth	2–3 mm	2.1 mm	2.6 mm	6.0 mm
Cross-sectional OCTA	Yes	No	Yes	Yes
Motion correction	Yes	No	No	Yes
Projection artefact removal	Yes	Yes	Yes	NA (no projection artefacts hence no need to remove)
Anterior segment function	Yes	No	No	No
Quantitative analysis	Yes	Yes	Yes	Yes
Comparative follow-up	Yes	No	Yes	Yes

All OCTA systems are based on Fourier-domain solutions, which includes spectral domain (SD) and swept-source (SS) systems. AngioVue (Optovue, Inc., Fremont, California, USA) and Angioscan (Nidek Co Ltd., Gamagori, Aichi, Japan) are SD OCTA systems, which uses SSADA and optical microangiography algorithm, respectively. On the other hand, Triton DRI-OCT (Topcon Corporation, Tokyo, Japan) and PLEX Elite Prototype 9000 (Carl Zeiss Meditec, Dublin, California, USA) are SS OCTA systems, which uses ratio analysis and complex microangiography, respectively. Images from AngioVue are typically smoother and clearer due to the combination of horizontal and vertical scanning and the use of the SSADA algorithm, which improves the signal-to-noise ratio of the flow detection [6, 8]. On the other hand, the field of view of Angioscan is larger (9 × 9 mm) than that of AngioVue (3 × 3 mm, 6 × 6 mm, 8 × 8 mm) and allows panoramic images (12 × 9 mm) to be taken. This can be useful when imaging the cornea, as cornea vascularisation often covers a large area, and thereby reducing the need for multiple scans [6]. In addition, Angioscan possess a montage software that splices adjacent OCTA images together to form a combined image. Although this feature is currently only optimized for the retina, future studies may eventually allow this technique to be used for the anterior segment [6]. However, the acquisition time for Angioscan is slightly longer than AngioVue as scanning speed is slower, which may lead to more motion artefacts from saccadic eye movements [6]. There have been studies comparing AngioVue and Angioscan. While it was found that image quality from both systems were comparable, the vessel density values cannot be compared [1, 8].

The swept-source OCTA system uses light source with significantly higher wavelength (1050 nm) than AngioVue (840 nm) and Angioscan (880 nm). This enables penetration to deeper layers of the eye [7]. However, as larger wavelengths result in lower resolution and lower signal strength in superficial layers, image enhancement software is necessary. In addition, swept-source OCTA systems have a much higher scanning speed (200,000 scans/sec) compared to AngioVue (70,000 scans/sec) and Angioscan (53,000 scans/sec), resulting in a wider field of view and better resolution. While all OCTA systems have in-built eye tracking systems that are designed for the posterior segment, the eye tracking system of PLEX Elite also performs well for the anterior segment, wherein scanning will stop when motion is detected. This can help reduce the amount of motion artefacts due to weak fixation, saccadic eye movement or poor patient cooperation. Recently, Akagi et al. showed that PLEX Elite successfully visualized intrascleral and conjunctival vessels [15].

### Optical coherence tomography angiography for the cornea

The healthy human cornea is avascular and transparent [2]. It is believed that the balance of angiogenic and anti-angiogenic factors is important to ensure this avascularity and is maintained by the inhibition of immune and inflammatory response [1, 12]. Corneal vascularisation is a pathological condition whereby the normal avascular cornea loses transparency due to the ingrowth of blood vessels [12]. It can result from diverse aetiologies, including chemical injury, chronic hypoxic conditions from contact lens use, limbal stem cell deficiency and infections such as herpes and trachoma [12, 14, 16]. Detecting and treating corneal vascularisation is critical as it can lead to adverse consequences, such as persistent inflammation, corneal oedema, scarring, significant loss in visual acuity or even blindness [16]. In addition, corneal vascularisation can cause disruption of the cornea’s immunologically privileged state, and thus increases the risk of graft rejection from corneal transplant procedures [1, 14]. Qualitative and quantitative assessments are needed for optimal disease monitoring, treatment planning and prognostic evaluation [4]. Studies have shown that OCTA is a promising method to evaluate corneal vasculature.

**Fig. 2:**
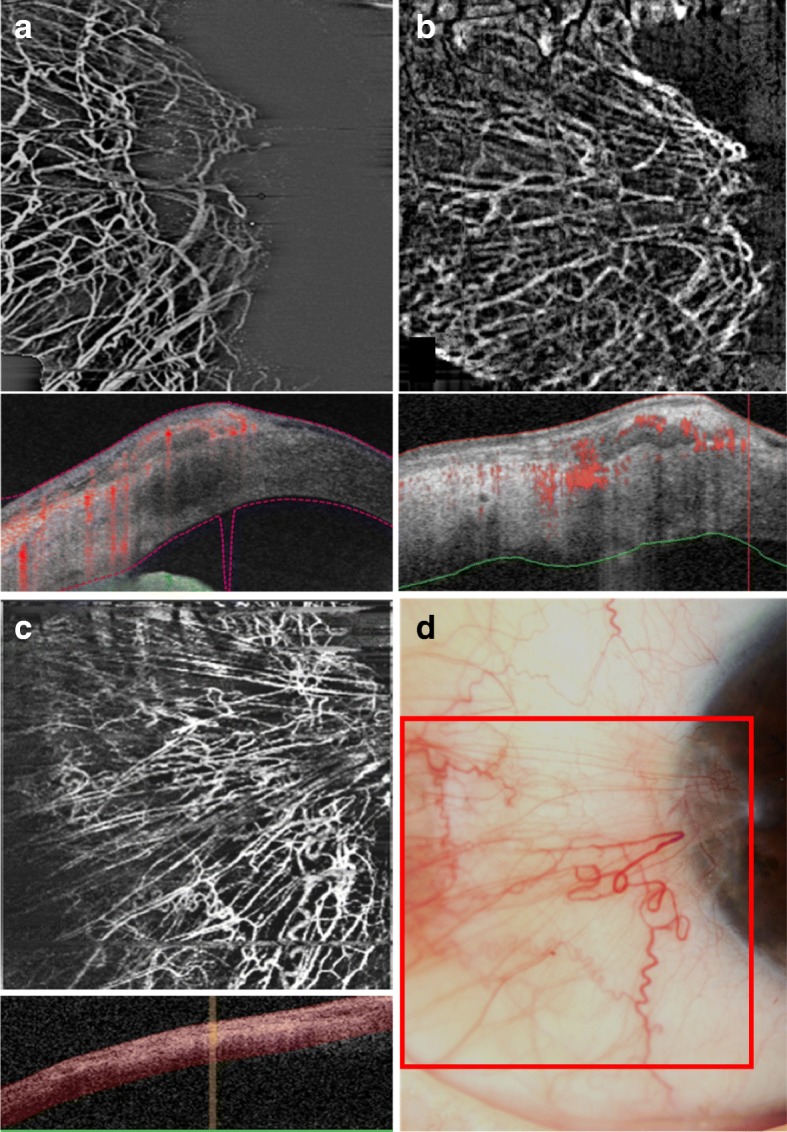
Examples of AS-OCTA scans of pterygium. Top: **a** PLEX Elite 9000 swept-source OCTA system, **b** AngioVue RT Vue XR Avanti OCTA system in the same eye. Bottom: **c** Angioscan RS-3000 Advance OCTA system and (**d**) corresponding slit-lamp photograph in another eye. The area bordered by the red square in the slit-lamp photo is matched to the Angioscan OCTA image

Although OCTA is not yet extensively used in clinics, it already has some useful clinical indications such as detecting vascularisation that are not visible due to reasons such as scarring [4]. Images of good quality and repeatability have been obtained for normal avascular corneas and abnormal corneal vascularisation due to herpetic keratitis, penetrating keratoplasty, bacterial keratitis, limbal stem cell deficiency and pterygium (Fig. 2) [14]. The *en face* function allows smart estimations of the depth and area of vascularisation, implying the possibility for follow-up at the exact region of interest, and pre- to post-treatment monitoring of progression and regression of the vascularisation [14]. This was illustrated in a study by Cai et al. on patients who underwent various treatments following graft rejection. It was found that OCTA was able to detect a significant decrease in the area of vascularisation three months post-treatment, which corresponded with colour photographs [12].

In addition, studies suggested that OCTA may be able to visualize early corneal vascularisation more clearly than SLP [4, 10]. Also, OCTA may reveal fine abnormal vessels that in cases with corneal opacification, vascularisation in the cornea periphery or anterior to the iris, would have gone undetected by SLP [4, 10]. In an animal study done on rabbits with induced corneal vascularisation, it was found that OCTA could capture small and regressed vessels that were not detected by SLP and better delineated than ICGA [13]. Other studies also found that images obtained by OCTA are comparable to that of ICGA and FA, although they were not conclusive if ICGA or OCTA detects larger vessel density [1, 8, 13]. It should also be acknowledged that images of the entire area of corneal vascularisation can only be acquired with ICGA and require multiple scans by OCTA due to the limited field of view [1]. Nonetheless, with further development of software and hardware, OCTA could prove to be an invaluable asset in the clinics and become the gold standard in assessing anterior segment vasculature.

**Fig. 3:**
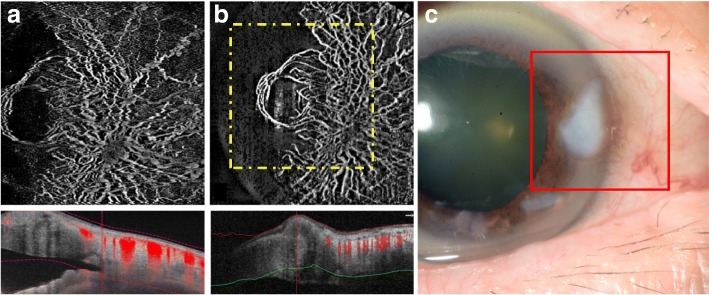
Examples of AS-OCTA scans in an eye with corneal vascularisation. Corneal vascularisation was clearer in the AS-OCTA images obtained using (**a**) PLEX Elite 9000 swept-source OCTA system, and (**b**) AngioVue RT Vue XR Avanti OCTA system compared to slit-lamp photography (**c**). The corresponding area imaged with slit-lamp photography and AngioVue OCTA system are matched (bordered by red and yellow squares, respectively)

With the combined structural and vascular information, OCTA can potentially aid diagnosis of corneal pathologies (Fig. 3) and pre-operative surgical planning such as determining the depth of feeder vessel diathermy before anterior lamellar keratoplasty for lesions with abnormal vessels, assessing effectiveness of new therapies for corneal vascularisation, and prognostication such as early limbal stem cell deficiency [3, 4].

### Optical coherence tomography angiography for the IRIS

Normal iris vasculature consists of a major arterial circle that is connected to the anterior and long posterior ciliary arteries, and a minor arterial circle found along the border of the pupil linked by radially oriented vessels within the iris stroma [5]. The role of the iris and its vasculature have been increasingly recognized in homeostasis of the anterior chamber and pathogenesis of some eye diseases, including glaucoma and cataracts [17]. It was postulated that iris vasculature studies can shed light on pathophysiology of developmental anomalies, degenerative diseases, diabetes microangiopathy, glaucoma and uveitis [5]. The earliest study done to investigate iris vasculature used FA and ICGA, but given their invasive nature, was limited in scope [18]. Hence, non-invasive OCTA has become an appealing alternative [5].

**Fig. 4:**
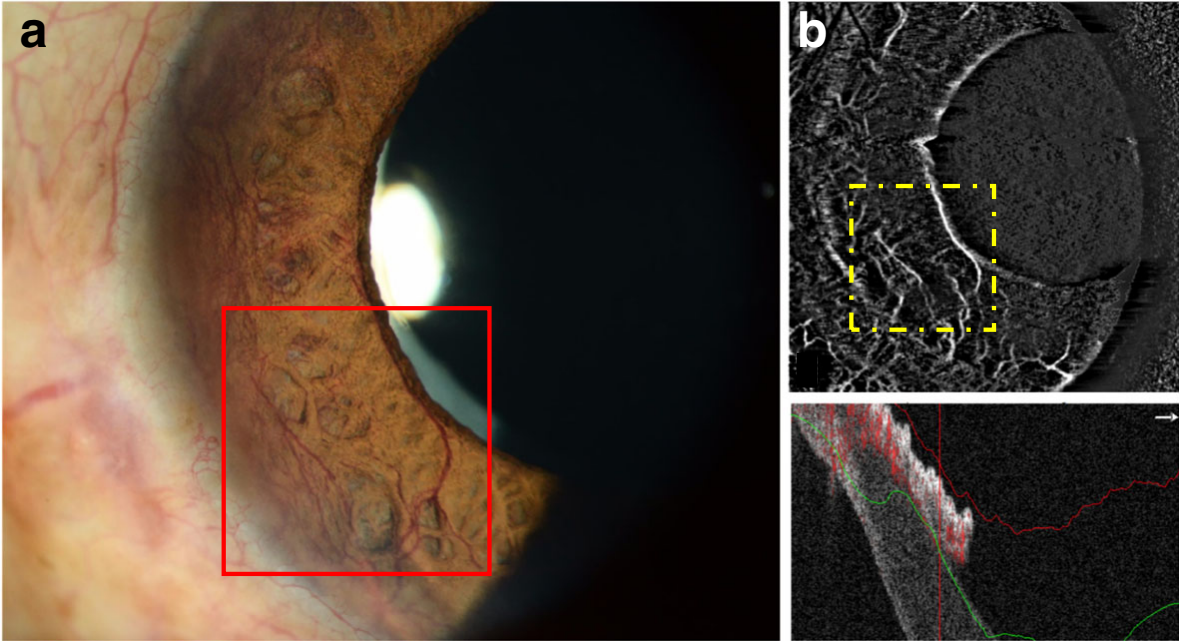
Example of AS-OCTA scans in an eye with neovascular glaucoma. Abnormal iris neovascularization (**a**) are delineated by the AS-OCTA system (**b**), however, some vessels on the iris are not clearly seen on the AS-OCTA scans. This may be due to poor segmentation, image artefacts or thresholding issues. The corresponding area imaged with slit-lamp photography and OCTA are matched (bordered by red and yellow squares, respectively)

While there have been few studies investigating the use of OCTA for iris vasculature, those studies provided interesting insights into its potential use. OCTA has been found to produce comparable images of differently pigmented healthy iris with FA, but with significantly more detail [5, 18]. However, as the number of visible vessels was found to be negatively correlated to iris pigmentation, the inability to image iris vasculature for densely pigmented iris remains a limitation for all current imaging techniques, including FA and ICGA. OCTA was also able to visualize the diseased iris, including presence of iris melanocytic tumours and iris neovascularisation (NVI; Fig. 4) [19, 20]. NVI develops secondary to a range of ocular diseases, including retinal vein occlusion, diabetic retinopathy, ocular ischemic syndrome and uveitis [19]. Early detection of NVI may allow for timely medical intervention before complications such as rubeotic glaucoma develop; OCTA was able to detect subclinical NVI that appeared in its early stages [19].

Iris melanomas are tumours that increase the risk of vision loss and metastatic disease. As there has yet to be an effective treatment for metastatic disease, it results in death in the majority of patients diagnosed with it [20]. Visual acuity might also worsen due to treatment of tumours, such as excisional surgery or radiation. Due to the downsides of these treatments, cases are usually observed until indicative of metastatic disease. One indicator of malignant transformation is the increase of intra-tumoral vascularity [20]. Furthermore, as these tumours are often asymptomatic, a non-invasive method to routinely image the iris vasculature will be helpful. In one study, OCTA demonstrated the ability to image hypervascularity of iris melanomas, which was consistent with reports done with FA [20]. The reduction of intra-tumoral vessel density was also observed in cases treated with radioactive plaques. However, OCTA was not able to visualize vessels in dense tumours even with higher wavelength light source [20]. Nonetheless, OCTA has demonstrated the ability to visualize melanocytic tumours and its vascularity in a non-invasive way which may be developed for future use.

OCTA is expected to play an important role in imaging of the iris vasculature in the future as the need for non-invasive methods gains more recognition. Apart from the applications mentioned above, OCTA may also be useful in the diagnosis of ischemic conditions in systemic disease, or vascular changes secondary to uveitis, hypertension, diabetic retinopathy or obstructive conditions [5, 21]. It can also be useful for assessing effects of anterior segment implants, such as iris supported phakic intraocular lenses [5]. However, some limitations of OCTA for iris imaging need to be addressed, including the inability to penetrate highly pigmented iris or dense tumours, its limited field of view to image the iris in a single scan and the lack of a tracking system to compensate for iris movement [5, 20].

### Optical coherence tomography angiography for sclera, EPISCLERA and conjunctiva

Very limited OCTA studies have been done on the sclera and conjunctiva [15]. Recently, one study revealed that OCTA successfully visualized intrascleral and conjunctival vessels, with a denser vasculature presented than conventional FA. While episcleral and conjunctival vessels have been imaged by other imaging modalities such as FA, non-invasive evaluation of vessels at a specific depth and imaging of intra-scleral vessels have been challenging [22]. On the other hand, OCTA has allowed for non-invasive depth-resolved imaging, thus overcoming these limitations [15]. The ability to image scleral and conjunctival vessels easily will be beneficial to understand conditions such as scleritis or uveitis, or the effect of the sclera and conjunctiva on glaucoma filtration surgery [15]. In the future, OCTA imaging may be used to intra-operatively assess the aqueous humour outflow pathways and episcleral venous outflow, and to evaluate bleb morphological features after glaucoma surgery [15].

### Current limitations of OCTA for the anterior segment

Current OCTA systems are designed specifically with the intention of imaging the posterior segment. Thus, adapting OCTA for the anterior segment have resulted in some limitations. Firstly, there is a need to adjust scanning protocols and to use anterior segment adaptor lens [4, 6]. Since the internal software of these systems are calibrated for the posterior segment, there can be non-parallel segmentation and artefacts caused by light scatter due to the cornea curvature, resulting in inaccurate vessel density calculations during depth-resolved analysis [1, 13].

Secondly, the in-built eye-tracking systems cannot be used for anterior segment to allow follow-up scans. Anterior segment OCTA (AS-OCTA) is unable to register patients and provide localization required for comparison of serial scans [2, 14]. While current studies on serial OCTA have shown that an image processing software for image analysis have helped manage this difficulty, an eye-tracking system designed for the anterior segment is still desirable, also because it helps to reduce motion artefacts considerably, which in turn improves image quality [3, 12]. Image artefacts are common in AS-OCTA scans. As AS-OCTA systems do not yet have motion correction for saccadic eye movement, these movements often result in motion artefacts [3]. Furthermore, vessels in the superficial layers can cause projection artefacts on the deeper layers as a result of multiple scattering. This can be misinterpreted by image analysis software as abnormal or additional vessels, resulting in inaccurate vessel density calculations. However, this problem can be mitigated by performing multiple scans and comparing these consecutive scans in *en face* function or correlating with images from other techniques such as SLP [4, 12]. In addition, with improvements in image analysis software, automated segmentation ability, better filtering techniques and threshold analysis, artefacts can be better managed [12].

Thirdly, AS-OCTA may not delineate deeper vessels in eyes with corneal opacities or dense iris pigmentation, or vessels in thick iris tumours [4, 5, 20]. The system also may have poorer detection of vessels with minimal flow since motion of erythrocytes are much slower in those vessels with small diameters and this may be below the level of detection [1]. This lower limit of detection is dependent on the A-scan rate of the OCT system. The faster the system, the lower the velocities that can be visualized. Since internal system algorithms of OCTA are optimized for the posterior segment with mainly transverse flows in those vessels, anterior segment vessels with axial flow may not be well detected [8]. This is related to the principle of OCT because flow that is parallel to the incident laser beam does not lead to a decorrelation signal.

### Future directions of optical coherence tomography angiography for the anterior segment

While adapting OCTA systems for anterior segment imaging poses many challenges, it is important to note that there is a lot of work being done in the industry to rapidly improve the hardware and software of AS-OCTA. Therefore, many of the limitations mentioned above may be addressed sooner rather than later.

Hence, despite the limitations, it is recognized that AS-OCTA possesses promising potential for clinical applications. The ability of OCTA to image vasculature in the anterior segment may allow its future application in diagnosis and monitoring of pathological conditions in the cornea, iris, conjunctiva and sclera, including studying ocular inflammatory diseases, assessing corneal vascularisation for graft rejection, anterior segment tumour vascularity, secondary or neovascular glaucoma, limbal stem cell deficiency, NVI and assessing episcleral venous flow in glaucoma [12, 13]. Furthermore, with structural information from OCT scans, OCTA can aid in treatment management or surgical planning, such as for planning of corneal transplantation surgeries in vascular lesions or scars [23]. The quantitative information about the depth of pathology makes OCTA useful for evaluating the effectiveness of intervention, such as subconjunctival vascularity associated with bleb morphology after trabeculectomy [2]. Lastly, OCTA may be used as an assessment tool in the future for prognostication of ocular surface diseases or immunological rejection from graft transplants [2, 8].

AS-OCTA imaging is a new field and there are still many areas that require fine-tuning. Software enhancements could be developed to improve image resolution, reduce artefacts and enhance the depth of field in the cornea [4, 24, 25]. With further upgrading in scanning speed, improved wide-field imaging OCTA and automated montage functions by the internal software will become more realistic [3]. Furthermore, artefacts due to non-parallel segmentation and the lack of a reliant eye tracker for AS-OCTA imaging can be avoided with developments in eye tracking and image registration [6, 13]. Image processing algorithms that can reduce projection, shadow and motion artefacts are also needed [1]. Automated programs which already exist for AS OCT may be further developed to include AS-OCTA segmentation in the future [26]. With the rapid advancement of technology, it may be a good opportunity to investigate the use of artificial intelligence to generate normative databases and perform analysis for relevant OCTA studies [3]. Further studies on animal models may also be useful for understanding treatment methods or evaluating the possibility of combining OCTA with current fluorescence angiography techniques using multi-modal approaches [1, 13].

## Conclusion

OCTA allows for rapid, non-invasive imaging of vasculature within the eye. While commercially available systems are designed and optimised for the posterior segment, current progress in adapting OCTA for anterior segment imaging have been promising. With further improvements to better optimise the software, OCTA for anterior segment imaging will be an achievable reality soon.

## Abbreviations

AS: Anterior segment
FA: Fluorescein angiography
ICGA: Indocyanine green angiography
NVI: Iris neovascularisation
OCT: Optical coherence tomography
OCTA: Optical coherence tomography angiography
SD: Spectral domain
SLP: Slit-lamp photography
SS: Swept-source
SSADA: Split-spectrum amplitude decorrelation angiography
